# The Interplay Between Viral Infection and Cell Death: A Ping-Pong Effect

**DOI:** 10.1155/av/5750575

**Published:** 2025-02-03

**Authors:** Alireza Nourazarian, Hadi Yousefi, Cigir Biray Avci, Behrouz Shademan, Emad Behboudi

**Affiliations:** ^1^Department of Basic Medical Sciences, Khoy University of Medical Sciences, Khoy, Iran; ^2^Department of Medical Biology, Faculty of Medicine, EGE University, Izmir, Turkey; ^3^Stem Cell Research Center, Tabriz University of Medical Sciences, Tabriz, Iran

**Keywords:** autophagy, programmed cell death (PCD), pyroptosis, viral infection

## Abstract

Programmed cell death (PCD) is a well-studied cellular mechanism that plays a critical role in immune responses, developmental processes, and the maintenance of tissue homeostasis. However, viruses have developed diverse strategies to bypass or manipulate the host apoptotic machinery to enhance their replication and survival. As a result, the interaction between PCD pathways and viruses has garnered increased interest, leading to many studies being published in recent years. This study aims to provide an overview of the current understanding of PCD pathways and their significance in viral infections. We will discuss various forms of cell death pathways, including apoptosis, autophagy, necroptosis, and pyroptosis, as well as their corresponding molecular mechanisms. In addition, we will show how viruses manipulate host PCD pathways to prevent or delay cell death or facilitate viral replication. This study emphasizes the importance of investigating the mechanisms by which viruses control the host's PCD machinery to gain insight into the evolutionary dynamics of host-pathogen interactions and to develop new approaches for predicting and managing viral threats. Overall, we aimed to highlight new research areas in PCD and viruses, including introduction of new targets for the development of new antiviral drugs to modulate the cellular apoptotic machinery and novel inhibitors of host cell death pathways.

## 1. Introduction

Viruses are the most common biological pathogens, causing diseases with significant morbidity and mortality worldwide. As obligate intracellular pathogens, viruses rely on the host cellular machinery to ensure their replication, survival, and dissemination [1, 2]. The possible interactions between viruses and host cells appear more complex than previously thought. These interactions provide a remarkable opportunity to improve our understanding of cell biology and the molecular mechanisms of pathogenesis [3]. Furthermore, these interactions have significant implications for cellular and viral evolution. Although viruses are obligate intracellular parasites, they must find a replication or survival niche within cells by evading cellular defenses [4]. The success of viral infections depends on their ability to exploit basic biological processes, such as immune responses and programmed cell death (PCD) mechanisms [5].

Cell death mechanisms have several properties, the modulation of which is critical for successful pathogenesis [6]. The death of an infected cell may help the host because it interrupts the replication cycle and releases the pathogen for killing by immune cells. However, viruses can induce cell death to facilitate their spread or killing the infected immune cells or inhibit these programmed death pathways to hide in the host cells from the immune cells [7]. For example, some viruses pursue a virulence strategy by causing cell death to eliminate host cells or other immune cells (Table 1) [34] or to hide from the immune cells (Table 2) [57, 58]. Our comprehensive review emphasizes the significance of cell death mechanisms in virus-host interactions. This review provides literature-based information suggesting that cell death mechanisms function like a ping-pong ball during viral infections. Gaining a deeper insight into host interactions can enhance our comprehension of the molecular mechanisms that drive viral pathogenesis and facilitate the development of more effective therapeutic strategies.

## 2. Common PCD Pathways

Apoptosis, autophagy, necroptosis, and pyroptosis are distinct forms of PCD that are activated by different stimuli or stresses to keep cellular homeostasis or to eliminate damaged or infected cells [37, 59]. The following is a brief description of these processes:

1. Apoptosis constitutes a highly controlled mechanism of PCD that eliminates unnecessary or damaged cells during development or tissue homeostasis [60]. It is characterized by a series of biochemical events, containing chromatin condensation, nuclear fragmentation, plasma membrane bursting, and cell shrinkage [61]. This leads to the formation of apoptotic bodies that are quickly eliminated by phagocytic cells. Unlike other forms of cell death, apoptosis does not trigger inflammation or cell lysis. Apoptosis can be induced through the extrinsic or intrinsic pathway. In extrinsic pathway, after viral genome detection by retinoic acid-inducible gene I (RIG-I) and toll-like receptor 3 (TLR3), Fas activation induces tumor necrosis factor (TNF)-related apoptosis and in intrinsic, IFN-regulatory factor 3 (IRF3) activation stimulates the expression of IFN and induces apoptosis through an interaction with Bax [62].
2. Autophagy is an adaptive process of cellular self-digestion that is activated in response to nutrient deficiency, oxidative stress, or organelle damage. This process entails the generation of double-membrane structures called autophagosomes, which encapsulate cytosolic constituents and facilitate their delivery to lysosomes for degradation and subsequent recycling [47]. Autophagy regulated by autophagy-related proteins (Atgs) and Beclin1 can promote cell survival by providing energy and nutrients during stress but can also lead to cell death if overactivated [43].
3. Necroptosis is a type of programmed necrosis triggered by activating death receptors or intracellular sensors that recognize nucleic acids or viral proteins. This process is characterized by forming a punctate cytoplasmic structure known as a necrosome, which activates the kinase RIPK3 and the pseudokinase MLKL [63]. These, in turn, trigger the rupture of the plasma membrane. This leads to the leakage of the cytoplasm and the release of damage-associated molecular patterns (DAMPs), which activate innate immune responses. Necroptosis is associated with inflammation and tissue damage [64].
4. Pyroptosis is a type of PCD that is highly inflammatory and is triggered by the activation of the inflammasome in response to infection, host injury, or other stressors that generate cytoplasmic danger signals. The process involves the formation of pores in the plasma membrane through the action of the pore-forming protein gasdermin D (GSDMD) [65]. This triggers the release of cytokines, including IL-1β and IL-18, as well as a type of cell lysis that results in the release of intracellular contents, including potentially harmful DAMPs, such as HMGB1. Pyroptosis is a process commonly associated with inflammation and defense against infection [66].

### 2.1. Apoptosis and Viral Infection

Apoptosis is a noninflammatory form of PCD. It is characterized by a reduction in cell size and morphological changes, such as nuclear condensation and shrinkage of the plasma membrane [67]. These changes are due to the degradation of cellular proteins by cellular enzymes, especially caspases. Both cellular extrinsic and intrinsic mechanisms can trigger apoptotic caspase activity, and the regulatory signaling events involved in these pathways are well-characterized [68, 69]. Viruses can hijack cell death pathways, as seen in one of the first discoveries by Jeurissen, who linked virus-induced apoptosis to disease in chickens. He showed that infection with the chicken anemia virus could trigger apoptosis in thymus cells [70]. Cellular antiapoptotic genes can transform lytic viral infections into persistent ones, which may explain the long-term persistence of alphaviruses in the brain. Alphaviruses, such as Sindbis virus or Chikungunya virus, infect target cells, particularly neurons in the brain. The viral genome is released into the host cell, and the viral replication machinery hijacks the cellular processes to produce new viral particles. By blocking the apoptotic process, the antiapoptotic genes such as Bcl-2, Bcl-xL, or IAPs (inhibitor of apoptosis proteins) allow the infected cells to survive and continue to support viral replication. This transformation from a lytic to a persistent infection enables the virus to evade the host's immune response and establish long-term residence in the brain [71]. Since the discovery of apoptosis, several experiments have been performed in laboratories [72].

Interestingly, apoptosis contributes to the release of viruses; therefore, inhibiting apoptosis could prevent viral pathogenesis. Virus-induced apoptotic cell death plays a critical role in host immunity and viral shedding; however, it also causes virus-induced tissue damage and contributes to disease progression [1]. In the viral infections with some large DNA viruses including members of the adenoviruses, baculoviruses, herpesviruses, and poxviruses, it has been demonstrated that virus interference leads to decreased apoptosis, while certain viral infections, such as those caused by the Ebola virus or human immunodeficiency virus (HIV-1), may increase apoptosis [73, 74]. Viruses have developed strategies to bypass apoptosis, the PCD process, by encoding various inhibitors that suppress both the intrinsic and extrinsic mechanisms of cell death initiated by the host [75, 76]. Some adenoviruses, herpes simplex viruses, poxviruses, and cytomegaloviruses (CMVs) encode proteins homologous to the cellular antiapoptotic Bcl-2 family [40]. The first viral homolog discovered for Bcl-2 was the adenoviral protein E1B-19K. This protein blocks host cell apoptosis and maintains viral replication by inhibiting proapoptotic members of the Bcl-2 family [77]. E1B-19K can protect cells infected with adenoviruses from cell death induced by different stimuli, including E1A-triggered activation of TNF-α, p53, Fas, and TRAIL [78].

Human CMV additionally encodes a viral apoptosis inhibitor localized to the mitochondria, referred to as vMIA that targets mitochondria to inhibit Bax and suppress cell death. In contrast, vMIA also counteracts cell death via the serine protease HtrA2/Omi (high-temperature requirement protein A2/Omi stress-regulated endonuclease), which allows infected cells to survive for several days and continue producing the virus [79].

Recent studies have identified a small mitochondrial-localized protein encoded by some CMVs, such as murine CMV open reading frame (ORF) m41.1, which acts as a viral suppressor of Bak oligomerization (vIBO). Furthermore, to inhibit apoptosis triggered by different stimuli, HCMVs possess more proteins, including pUL38, IE1(491a), UL36, IE2(579aa), and viral RNA (vRNA) beta2.7 [80]. This RNA is associated with mitochondrial respiratory Complex I, which helps to maintain ATP production late in infection and prevents cell death caused by mitochondrial toxins [81]. Epstein–Barr virus (EBV) can also inhibit apoptosis through the multifunctional viral protein LMP-1 [82].

This process depends on the upregulation of Bcl-2 within the host cell and the upregulation of cellular antiapoptotic proteins such as A20 and bfl-1 [82]. In Hepatitis B virus (HBV), the HBV-X protein (HBx) plays a role in viral replication [83]. In addition, HBx has shown to play an oncogenic role in animal models and enhances the sensitivity of cells to apoptosis induced by TNF-α. The most severe symptom of poliovirus (PLV) infection is poliomyelitis paralysis [84]. This severe condition leads to flaccid paralysis due to caspase-dependent apoptosis of neurons. Similar to HBx, the PLV viroporin 2B is localized to the mitochondria, induces perinuclear redistribution of these organelles, and modifies their morphology. This suggests that 2B may have proapoptotic effects by directly facilitating mitochondrial membrane permeabilization (MMP) [85, 86].

HIV-1 protease promotes apoptosis and viral replication by cleaving and inactivating Bcl-2, leading to oxidative stress and activation of NF-κB, a transcription factor needed for HIV-1 enhancer activation. In contrast, apoptosis in virus-infected cells can be initiated either by activating the immune response or by introducing viral suicide genes that promote host cell death. Notably, considerable cell death during HIV-1 infection is largely due to the direct cytopathic effects of the virus on infected peripheral blood mononuclear cells in individuals with AIDS [76]. The HIV-1 envelope glycoprotein complex (Env) has a very strong ability to induce apoptosis. However, the apoptogenic effect of Vpr can be seen when using genetically modified HIV-1 strains called pseudotyped viruses, where the Env gene has been replaced with nonapoptogenic Env proteins from other viruses [87].

Hepatitis C virus (HCV) nonstructural protein 3 (NS3) is a part of the protease complex, and it is thought to induce Caspase-8-mediated apoptosis independently of its enzymatic activity [88]. Another disease, human hemorrhagic fever syndrome, is caused by the highly pathogenic Ebola virus and is associated with increased cell death. The disease triggered by the virus is characterized by an increase in inflammatory cytokines in plasma, including IL-1β, IL-6, and TNF-α [89].

**Figure 1:**
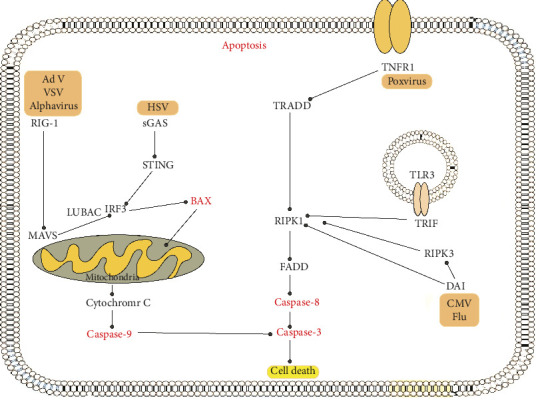
Apoptosis modulation by some of viruses through various host factors.

Apoptosis serves as the main mechanism responsible for stress-induced cell death. However, alongside apoptosis, viral infections can elicit various forms of cell death or a combination of these mechanisms. For instance, infection of motor neurons with the Sindbis virus results in cell loss in the spinal cord via necrosis while simultaneously inducing classical apoptosis in cortical neurons of the brain. This indicates a cell-type-specific response to the same viral agent [90]. A summary of the host-virus interactions is illustrated in Figure 1.

### 2.2. Autophagy and Viral Infection

Autophagy is one of the most important homeostatic processes. It is a self-eating mechanism by which host cells degrade cytoplasmic materials, such as protein clumps, and eliminate damaged organelles [91, 92]. In addition, the autophagy system provides selective transport of cytosolic contents, such as microorganisms, to lysosomes for degradation (xenophagy) and activation of innate and adaptive immunity by delivering viral nucleic acids and antigens to endolysosomes. However, several viruses use the host autophagic machinery to support their replication by modulating autophagy [91].

When a viral infection begins, one of the host's responses is to eliminate the viruses by xenophagy. Many viruses can be cleared in vitro by xenophagy. In vivo studies suggest that autophagy genes protect the host from viral pathogens such as HSV-1, Sindbis, and Chikungunya [91–93]. Specifically, host genes that modulate autophagy are associated with susceptibility to infection [93].

In a 1998 study, Beclin1 induced expression of the autophagy genes (Atgs) in neurons protecting the host from lethal alphavirus encephalitis. This was the first evidence that autophagy could effectively treat microbial diseases. Therefore, pharmacological agents that upregulate autophagy could be used as therapeutic agents for treating viral infections [39, 94]. To confirm this function, vitamin D treatment was reported to inhibit HIV replication in human macrophages via the autophagy machinery [95, 96].

Some viruses have different mechanisms for autophagic degradation. Many viruses have developed strategies to evade cell elimination via autophagy. For example, some herpes viruses, including herpes simplex virus type 1 (HSV-1), human CMV (HCMV), Kaposi's sarcoma-associated herpes virus (KSHV), and a poxvirus named Molluscum contagiosum virus (MCV), can effectively bypass autophagy [97]. HSV-1 can suppress autophagy via the viral proteins ICP34.5 and Us3, which inhibit Beclin-1 (a necessary protein for autophagosome formation and initiation of autophagy) [39]. On the other hand, DNA viruses such as varicella zoster virus (VZV), EBV, adenovirus, human papillomavirus 16 (HPV16), human parvovirus B19, simian virus 40 (SV40), and HBV can activate parts of the autophagy pathway and use them to increase viral replication. In the life cycle of an adenovirus, for example, cell death triggered by autophagy helps the virus to release particles [98, 99].

Other RNA viruses, including coxsackievirus B4 (CVB4), VSV, dengue virus-2 (DENV2), PLV, rotavirus, HCV, and influenza virus A, induce autophagy but inhibit autophagosome–lysosome fusion. Induction of autophagy provides cell membranes for RNA replication in viruses such as PLVs and HCV [100]. The viral M2 protein of influenza A virus (IAV) inhibits autophagosome–lysosome fusion, blocking the presentation of IAV proteins by MHC antigen and reducing the host's immune response [101].

Experimental investigations have demonstrated that genetic silencing of the autophagy proteins Beclin-1 or Atg7 in HCV-infected human hepatocytes results in caspase-dependent apoptosis. Conversely, the inhibition of Atg4b is linked to cytoplasmic vacuolization and the demise of HCV-replicating human hepatoma cells [102]. Similarly, infection with the Japanese encephalitis virus (JEV) led to increased caspase activation and cell death in cells deficient in Beclin-1 or Atg5 [103]. Notably, both HCV- and JEV-infected cells exhibited elevated production of interferon-α and interferon-β when autophagy was suppressed, suggesting that autophagy plays a role in modulating the antiviral interferon response [102, 103].

These findings indicate that HCV and JEV promote autophagy to sustain a persistent infection by inhibiting host cell death and disrupting the innate immune response. Similar results were observed in cells infected with flaviviruses or γHV68. Furthermore, the suppression of flavivirus NS4A protein-dependent autophagy through Beclin-1, Class III PI3K, or the inactivation of Atg5 led to increased mortality of infected epithelial cells and fibroblasts. This underscores the protective role of the autophagic response during flavivirus infections [104].

**Figure 2:**
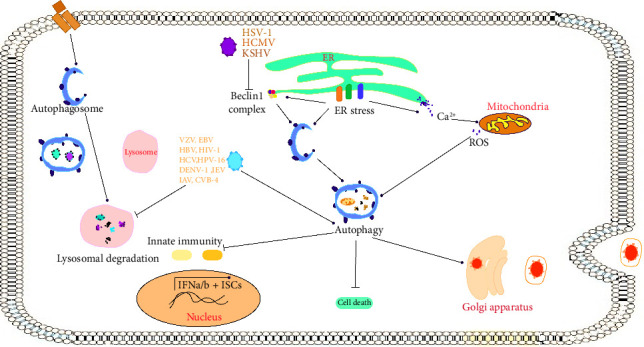
Autophagy modulation by some of viruses through inhibition or induction of different pathways.

The research has demonstrated that influenza viruses can trigger cytotoxic autophagy in vivo, evidenced by the decreased lung damage and lower mortality rates in mice infected with H5N1 when cytotoxic autophagy was pharmacologically inhibited or silenced through RNA interference targeting Beclin-1 [42]. Similarly, mice with selective deletion of Atg5 in the pancreas showed decreased pancreatic pathology after coxsackievirus B3 (CVB3) infection. Suppression of autophagy was associated with increased cell viability and decreased HIV-1 and CVB3 replication and EV71 viral particle release. This increases the possibility that autophagy is indirectly related to cell death through the promotion of viral replication [41]. A summary of the host-virus interactions is illustrated in Figure 2.

Autophagy is associated with cell death and can function as a defense mechanism that does not maintain cell viability, a removal mechanism that clears cellular debris from a cell that is already destined to be killed, or a pathway that supports other forms of cell death such as apoptosis, necroptosis, or pyroptosis. The recent research into the genetic modification of autophagy has yielded evidence supporting the role of autophagy in particular cell death pathways [105]. However, whether this occurs independently or in conjunction with apoptotic or necrotic death pathways is still unclear.

### 2.3. Necrosis and Viral Infection

Necrosis is a form of cell death marked by morphological changes, including cell rounding, organelle swelling, DNA fragmentation, and disruption of the plasma membrane. Changes in the plasma membrane lead to the release of intracellular contents, cell lysis, and the presence of DAMPs that can influence inflammatory responses [106]. Necrosis is defined as necroptosis that depends on the kinase activity of receptor-interacting protein kinase 1 (RIPK1) and RIPK3. Various types of necrotic cell death can be categorized according to the mechanisms that initiate them. Much of our understanding of necrosis comes from the study of TNF-α-induced necroptosis [107]. Cells infected with the Semliki Forest virus, human herpes virus 7, murine polyomavirus, and PLV exhibit two key features of cell death: apoptotic and necrotic changes [108]. Necrotic death predominates in early infections, as in the case of polyomavirus-infected cells, while apoptosis is seen at later stages. SV40-infected primary cortical neurons show both apoptotic and necrotic changes [109]. As a result, neurons from the same brain region may respond differently to viral infections, which could be due to either the inherent heterogeneity of the cortical neuron population or differential viral exposure [109, 110].

### 2.4. Necroptosis and Viral Infection

Necroptosis is a type of PCD known as regulated necrosis, differing from apoptosis in both morphology and biochemistry. This type of PCD is activated when apoptosis fails to respond to TNF-α. In contrast to apoptotic cells, which are cleared by macrophages or adjacent cells, necrotic cells release signals that provoke inflammation and worsen cell damage [111]. Current knowledge suggests that programmed necrosis is closely linked to the pathophysiology of various diseases and has shown to have the potential to activate an innate immune response against viral infections [110].

Biological processes, such as immunology and differentiation, can trigger regulated cell death. In addition, extrinsic necroptosis serves as a defense mechanism for the host against microbial infections. Various viruses, including adenoviruses, poxviruses, and herpes viruses, employ different strategies to evade the host's apoptosis machinery and promote replication [112]. Despite the threat of viral invasion, host cells have various defense mechanisms to counteract such attacks. For example, vaccinia viruses (VVs) are known to carry a Caspase-8 suppressor that effectively prevents apoptotic cell death during infection [105]. Consequently, cells undergo an alternative form of PCD called necroptosis.

Consistent activation of the necroptosis process is vital to trigger the innate immune response. Necroptosis not only eliminates virus-infected cells but also transmits danger signals from host cells to the external environment [105]. In addition, necroptosis of T-cells plays a crucial role in regulating the proliferation and survival of antigen-activated T-cells. Caspase-8 functions as a negative modulator of necroptosis and supports the activity of T-cells under normal biological circumstances [113]. In mice, the absence of Caspase-8 results in a lack of immune response of T-cells to murine hepatitis virus infection. RIPK3 is a crucial serine/threonine kinase that acts as a virtual adapter in necroptosis [52].

Furthermore, RIPK3 must maintain a “RIP homotypic interaction motif (RHIM)” at its C-terminus. In their quest to identify additional viruses capable of inducing necroptosis, Schock et al. discovered that the Sendai virus triggers necroptosis when combined with the pan-caspase inhibitor zVAD-fmk. This study shows that activation of the RNA sensor RIG-I can lead to regulation of necroptosis by the Sendai virus [46, 52].

Herpesviruses employ the necroptotic pathway through various RHIM adapters, such as ICP6 from HSV-1, M45 from MCMV, and ICP10 from HSV-2. These viral suppressors circumvent the interaction and activation of ZBP1 and RIPK3 [114]. However, without viral RHIM inhibitors, ZBP1 detects vRNA and triggers the binding and activation of RIPK3. The role of viral RHIM adapters in regulating necroptosis varies between host species. For example, although ICP6 and ICP10 suppress necroptosis in human cells, they trigger RIPK3-dependent necroptosis in mouse cells. This difference in response probably is due to the fact that the virus is a human-specific virus and not adapted for mice and therefore it is showing different responses [46].

**Figure 3:**
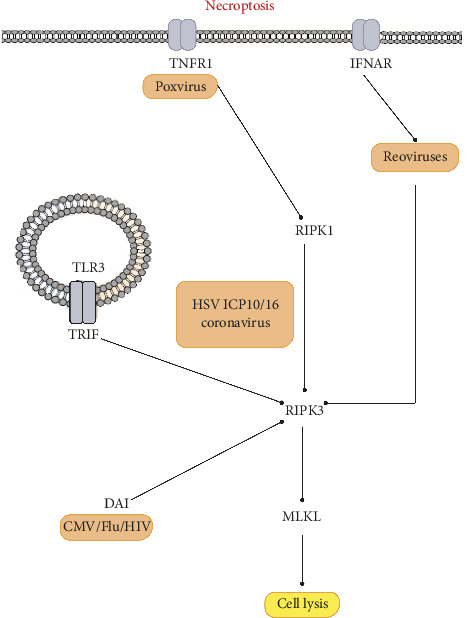
Necroptosis modulation by different viruses in different stages.

Large-DNA viruses often employ a caspase-targeting strategy to prevent their inhibition, leading to viral persistence and evasion of the host's immune system [53]. For example, poxviruses encode serpins that obstruct Caspase-1 and Caspase-8. The inhibition of Caspase-8 by poxviruses triggers a natural signal of necroptosis [56]. In contrast to herpesviruses, the IAV utilizes this mechanism to trigger ZBP1/RIPK3-dependent necroptosis [115]. Interestingly, TNF-α can induce necroptosis in cells infected with both the VV and its strain used to vaccinate people (modified Vaccinia Ankara) [116]. Apoptosis and necroptosis are two distinct host defense mechanisms that offset each other in response to viruses that have developed countermeasures [50]. Programmed necrosis can be seen as a “back-up plan” for extrinsic apoptosis. Controlled necroptosis, which involves the activation of caspases by Caspase-8, is crucial for development of a successful defensive mechanism [117]. It exerts selection pressure on viruses, forcing them to acquire suppressors while simultaneously triggering Caspase-8. When Caspase-8 activity or RIP1 polyubiquitination is inhibited, receptor-interacting protein (RIPK3) becomes active [112]. A summary of the host-virus interactions is illustrated in Figure 3.

### 2.5. Pyroptosis and Viral Infection

Pyroptosis is a type of PCD that differs from other forms of cell death in both morphology and mechanism. A hallmark of pyroptosis is its dependence on Caspase-1, which plays a critical role in mediating the mechanism of cell death [118]. Interestingly, Caspase-1 is not directly involved in apoptosis. As a result, mice deficient in Caspase-1 do not present any abnormalities. The recent research has found GSDMD as a mediator of pyroptosis, a form of PCD that triggers inflammation [119]. Pro-inflammatory caspases such as Caspases 1, 11, 4, and 5 cleave GSDMD, which generates a nonselective pore in the plasma membrane, ultimately leading to pyroptosis. During the activation of pyroptosis, Caspase-1 initially triggers the release of proinflammatory cytokines, including interleukin-1β (IL-1β) and IL-18 [120]. Second, Caspase-1 and Caspase-11 can induce pyroptosis. Cell lysis, the release of cytokines, such as IL-1β and IL-18, and the release of cellular contents increase the recruitment of inflammatory cells, leading to the activation of immune cells and the increased production of cytokines [121].

During pyroptosis, Caspase-1 is activated by the inflammasome, and its adapter protein named PYCARD also known as ASC, which is also involved in apoptosis and happens to have both, a PYD domain and a CARD domain. The formation of the inflammasome can be triggered by various protein activators, such as absent in Melanoma 2 (AIM2), NOD-like receptor protein (NLRP), or RNA sensor retinoic acid-inducible Gene-I (RIG-I). The AIM2 inflammasome is activated nonspecifically by cytosolic DNA from viruses, such as murine CMV (MCMV) and VV [122]. AIM2 has a HIN domain that can bind to double-stranded DNA (dsDNA). When MCMV DNA is present in the cell's cytoplasm after the virus has uncoated, the HIN domain of AIM2 can recognize and bind to this viral dsDNA. This binding of AIM2 to the MCMV dsDNA triggers the activation of the AIM2 inflammasome. The AIM2 protein then recruits the adapter protein ASC, leading to the assembly of the inflammasome complex in the cytoplasm. The formation of the AIM2 inflammasome then leads to the activation of Caspase-1, which in turn cleaves and activates proinflammatory cytokines such as IL-1β and IL-18, as well as the protein GSDMD that induces pyroptotic cell death [123].

Pyroptosis plays a vital role in HIV infection. Both DNA and RNA viruses can trigger pyroptosis by activating the inflammasome. The interferon-gamma-inducible protein Ifi-16 (IFI16), a DNA sensor, detects viral DNA produced during HIV-1 infection in the cytosol of macrophages or CD4+ T-cells [124]. This recognition by IFI16 limits viral replication in human macrophages and promotes pyroptosis in CD4+ T-cells within lymphoid tissues. RNA viruses that activate the AIM2 inflammasome include Chikungunya and West Nile viruses (WNV) [125]. Other RNA viruses that stimulate NLRP inflammasomes are influenza, hepatitis C, and dengue virus. The RIG-I inflammasomes are triggered by VSV and encephalomyocarditis virus [126]. Once these inflammasomes form, they lead to the activation of Caspase-1 through Caspase Recruitment Domain Family Member 16 (CARD16) [127]. When Procaspase-1 is chopped and converted to its activated shape, the p20 subunit of Caspase-1 activates the inflammatory cytokines IL-18 and IL-1β, which ultimately trigger pyroptosis [44].

**Figure 4:**
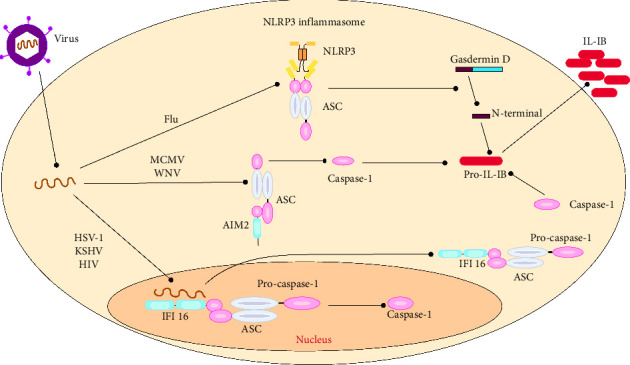
Pyroptosis modulation by some of viruses through different pathways and various cellular factors.

A study discovered that pyroptosis is induced in the preliminary stages of EV71 and CVB3 infection by activating Caspase-1 and secreting IL-1β and IL-18 [44]. Inhibiting pyroptosis reduced the inflammatory responses of virus-infected mice and decreased viral replication in both the CNS and myocardium [128]. Another study showed that HCV and DENV-2 infections could induce pyroptosis in endothelial cells (ECs), increasing membrane permeability. These findings provide insight into pyroptosis as an alternative form of PCD and the activation of Caspase-1 and -4 during DENV infection. Inflammatory caspases play a crucial role in innate immunity by responding to cytosolic signals and eliciting dual responses [54]. Pyroptosis of infected cells helps effector cells of the innate immune system eliminate pathogens and intracellular replicating pathogens. However, viruses manipulate pyroptosis to escape the immune system and replicate in infected cells [129]. A summary of the host-virus interactions is illustrated in Figure 4.

## 3. Future Perspective

PCD plays a crucial role in various biological systems, such as immune responses, development, and tissue homeostasis. Unfortunately, viruses have discovered ways to either bypass or manipulate the host PCD machinery for their own survival and replication, as shown by recent studies [130]. Viruses have devised various strategies to evade or manipulate the host PCD machinery, such as altering host apoptotic pathways, controlling autophagy-mediated cell death, and promoting forms of regulated necrosis, including necroptosis and pyroptosis [45]. To improve our understanding of the interplay between PCD and viruses, future studies should focus on elucidating the molecular mechanisms involved and finding novel therapeutic targets for viral infections. One promising area of research involves the development of antiviral drugs that can modulate the apoptotic PCD machinery, potentially halting viral replication [130, 131]. Another promising avenue of the research is the discovery of novel viral inhibitors that interfere with PCD signaling pathways in the host. These inhibitors could serve as the basis for new antiviral strategies.

A crucial perspective to consider is the influence of host-pathogen coevolution on viral infections. By examining how viruses alter host PCD machinery, researchers gain insight into the evolutionary dynamics of host-pathogen interactions. These investigations have the potential to produce innovative approaches to predict and manage emerging viral threats.

## 4. Conclusion

In summary, the relationship between PCD pathways and viruses is complex and dynamic. Depending on their replication or survival needs, viruses have developed different strategies to manipulate the host PCD machinery and promote or prevent cell death. This interaction between viruses and PCD pathways has opened new avenues for therapeutic development and provides valuable insight into the host-pathogen coevolution. The various types of PCD have different molecular mechanisms and functional outcomes. Viruses have shown to modulate these signaling pathways by various mechanisms, such as targeting upstream signaling pathways or inhibiting key effector molecules. Manipulation of PCD signaling pathways in the host can lead to antiviral strategies, including drug development or immunomodulation.

In addition, emerging areas of research, such as the identification of new antiviral targets and the study of the coevolution of host-virus interactions that affect PCD, suggest a promising future. Further research on PCD pathways and viruses could lead to a deeper understanding of these complex relationships.

## Figures and Tables

**Table 1:** Cell death mechanisms' activation by different viruses and virus–host protein interactions.

PCD types	Virus	Viral protein	Host target	Effect on PCD	Ref
Apoptosis	HBV	HBx	P53	Modulation of P53	[8]
	HCV	NS4A and core	TNFR	Association with TNFR	[9]
	HIV	Vpr, E, Tat, and Nef	Fas L, ANT	Increase Fas L and interaction with ANT	[10]
	HPV	E7	P53	Activation of P53	[11]
	IAV	PB1-F2	P53	Activation of P53	[12]
	Coronaviruses (SARS-CoV-2, SARS-CoV, and MERS-CoV)	S, ORF3a, and M	Fas	Activation Caspase 8 and 9	[13]
	Flaviviruses	NS2B/NS3	TNFR	Activation Caspase 3	[14]
	VSV	M	TNFR	Association with TNFR	[15]
	Adenovirus	E1A	P53	Activation of P53	[16]

Autophagy	HBV	HBx	Beclin1-VPS34	Activation of Beclin1	[17]
	HIV	Nef, Tat, E, and Gag	Beclin1-NFkB	Activation of Beclin1	[18]
	HCV	NS4B/NS5A/Core	IRGM-Beclin1	Activation of Beclin1	[19]
	HPV	E5/E6/E7	Beclin1-P62-LC3	Activation of LC3 and Beclin1	[20]
	EBV	LMP1-LMP2	Beclin1-PI3K/AKT/mTor	Activation of Beclin1	[21]
	KSHV	vCyclinD-vFLIP	AMPK-ATG3	Activation of Atg cascade	[22]

Necroptosis	HSV	ICP6/ICP10	RIP1 and 3-MLKL	Activation of RIP1 and RIP3 and increase MLKL	[23]
	IAV	IE3	RIP1	Activation of RIP1	[24]
	HIV	E, Tat	RIP1 and 3	Activation of RIP1 and RIP3	[25]
	Reoviruses	NSP4	RIP1 and HMGB1	Activation of RIP1 and RIP3 and increase HMGB	[26]
	IAV	PB1-F2	RIP1 and 3	Activation of RIP1 and RIP3	[27]
	Coronaviruses (SARS-CoV-2, SARS-CoV, and MERS-CoV)	NSP13	RIP1 and 3-ZBP1	Activation of RIP1 and RIP3	[28]

Pyroptosis	HIV	Nef, Tat	NLRP3	Activation of NLRP3	[29]
	Rabies	E4	Caspase 1-GSDMD	Activation of Caspase1-GSDMD pathway	[30]
	IAV	NS1	Galectin	Activation of galectin and activation of NLRP3	[31]
	Enteroviruses	NSP4	Caspase 1-GSDMD	Activation of Caspase 1-GSDMD pathway	[32]
	Flaviviruses	NS5	NLRP3	Activation of NLRP3	[33]

**Table 2:** Cell death mechanisms' inhibition by different viruses and virus–host interactions.

PCD type	Virus	Viral proteins	Targets	Effect on PCD	Ref
Apoptosis	HSV	ICP6-ICP10	Caspase 8	Caspase inhibition	[23]
		ICP22	Caspase 3 and P53	Caspase inhibition	
		ORF71	Bcl2	Bcl2 homolog	
		US3	Caspase 3/8,NF-kB, IP3K, and Bcl2	Inhibits caspase, increase PI3K/AKT, and inhibit NF-kB	
		US5	Caspase 8	Caspase inhibition	
		US6	Fas	Inhibition of TNF/Fas L	
		US8	BIM	Inhibition of BIM	
	HCMV	M36/UL36	Caspase8	Caspase inhibition	[35]
	KSHV	vFLIP	Caspase8	Caspase inhibition	[36]
	Adenovirus	E1B-19K	P53	Caspase inhibition	[37]
	HPV	E6	BAK	Inhibition of TNF/Fas L	[11]
	Poxviruses	SPI1/2/3	Caspase 8	Caspase inhibition	[38]
		CrmA-E	TNF	Mimics TNF	
		E3	PKR	PKR inhibition	

Autophagy	HSV	ICP34.5	Beclin1	Autophagy inhibition	[39]
		US3	mTORC/Beclin1	Autophagy exhaustion	
	HCMV	TRS1	Beclin1	Autophagy inhibition	[40]
		IRS1	Beclin1	Autophagy inhibition	
	EBV	BLF1	P62	Decreases its ubiquitination	[21]
	KSHV	vFLIP	Beclin1	Autophagy inhibition	[22]
	Picornavirses	3C	Atg5/Atg12	Degradation of Atgs	[41]
	IAV	M2	LC3	Relocalization to plasma membrane	[42]
	HBV	HBx	Unknown	Autophagy inhibition	[43]
	HIV	E	Beclin1	Autophagy inhibition	[18]
	CB3	2A	P62	Cleaves P62	[44]
	Coronaviruses (SARS-CoV-2, SARS-CoV, and MERS-CoV)	ORF3a/ORF7a	VPS39/UVRAG	Prevents SNARE assembly	[45]

Necroptosis	HSV	ICP6-ICP10	RIPK1-3	Inhibition of MLKL	[46]
	MCMV	M45	RIPK3	Inhibition of RIPK	[47]
	EBV	LMP1	RIPK1	Poliubiquitination of RIPK	[48]
	IAV	Hemagglutinin	RIPK1	Inhibition of RIPK	[27]
	Picornaviruses	3C	RIPK3	Cleavage of RIPK	[49]
	Poxviridae	E3	ZBP1	Inhibition of ZBP1	[50]
		vIRD	RIPK1	Cleavage of RIPK	
		vMLKL	RIPK1	Block sequestration of MLKL	

Pyroptosis	Coronaviruses (SARS-CoV-2, SARS-CoV, and MERS-CoV)	NSP4/NSP5	Gasdermin D	Cleavage of gasdermin D	[51]
		N	Gasdermin D	Cleavage of gasdermin D	
	Picornaviridae	3Cpro	Gasdermin D	Cleavage of gasdermin D	[52]
	KSHV	ORF63	NLRP3-ASC	NLRP3 inhibition	[20]
	EBV	miRNA-BART15	NLRP3-ASC	NLRP3 inhibition	[53]
	IAV	NS1	NLRP3-ASC	NLRP3 inhibition	[54]
	Paramixoviridae	V	NLRP3-ASC	NLRP3 inhibition	[52]
	Papillomavirus	E7	IFI16	Degradation of IFI16	[55]
	Poxviridae	M13L	NLRP3-ASC	Inhibition of inflammasome	[56]
		PYD		Inhibition of inflammasome	
		V		Inhibition of inflammasome	

## Data Availability

The data are included in the text, tables, figures, and referenced in the article.
